# Identification of Additive–Epistatic QTLs Conferring Seed Traits in Soybean Using Recombinant Inbred Lines

**DOI:** 10.3389/fpls.2020.566056

**Published:** 2020-12-10

**Authors:** Meng Li, Lingling Chen, Jian Zeng, Muhammad Khuram Razzaq, Xianchao Xu, Yufei Xu, Wubin Wang, Jianbo He, Guangnan Xing, Junyi Gai

**Affiliations:** Soybean Research Institute, MOA National Center for Soybean Improvement, MOA Key Laboratory for Biology and Genetic Improvement of Soybean (General), State Key Laboratory for Crop Genetics and Germplasm Enhancement, Jiangsu Collaborative Innovation Center for Modern Crop Production, Nanjing Agricultural University, Nanjing, China

**Keywords:** soybean, seed weight, seed shape, high density genetic map, QTL mapping, joint QTL-segment, candidate gene

## Abstract

Seed weight and shape are important agronomic traits that affect soybean quality and yield. In the present study, we used image analysis software to evaluate 100-seed weight and seed shape traits (length, width, perimeter, projection area, length/width, and weight/projection area) of 155 novel recombinant inbred soybean lines (NJRISX) generated by crossing “Su88-M21” and “XYXHD”. We examined quantitative trait loci (QTLs) associated with the six traits (except seed weight per projection area), and identified 42 additive QTLs (5–8 QTLs per trait) accounting for 24.9–37.5% of the phenotypic variation (PV). Meanwhile, 2–4 epistatic QTL pairs per trait out of a total of 18 accounted for 2.5–7.2% of the PV; and unmapped minor QTLs accounted for the remaining 35.0–56.7% of the PV. A total of 28 additive and 11 epistatic QTL pairs were concentrated in nine joint QTL segments (JQSs), indicating that QTLs associated with seed weight and shape are closely related and interacted. An interaction was also detected between additive and epistatic QTL pairs and environment, which made significant contributions of 1.4–9.5% and 0.4–0.8% to the PV, respectively. We annotated 18 candidate genes in the nine JQSs, which were important for interpreting the close relationships among the six traits. These findings indicate that examining the interactions between closely related traits rather than only analyzing individual trait provides more useful insight into the genetic system of the interrelated traits for which there has been limited QTL information.

## Introduction

Soybean (*Glycine max* [L.] Merr.) is widely cultivated and consumed, accounting for 70% of protein meal and 28% of vegetable oil sources globally in 2018 (SoyStat, 2019). As yield is the most important target trait for improvement of this crop, its closely related trait – seed size including seed weight and shape (volume) – have been widely investigated (Fang et al., 2017; Yu et al., 2017). Seed size, which is measured as 100-seed weight (100-SW), is a fitness trait that is critical for adaptation to a particular environment (Tao et al., 2017). Seed shape (volume) traits have also been the focus of improvement by farmers and soybean breeders (Garg et al., 2017); round seed varieties are more suitable for sowing and mechanical handling, and large-seed soybeans are more attractive for direct consumption (e.g., as edamame) while small-seed varieties are consumed as sprouts. Thus, 100-SW and seed shape not only affect production and processing, but also influence growers’ preferences in the propagation of a cultivar (Maughan et al., 1996).

While 100-SW of soybean can be easily measured, seed shape has not been well studied because the traits are ill-defined and their measurements are tedious and inaccurate. Imaging technology has recently been applied to the study of crop phenotypes (Price et al., 2011). For instance, a computer image-based software has been developed that can accurately measure soybean seed morphology traits including seed length (SL), seed width (SW), seed perimeter (SP), and seed projection area (SA) (Ding et al., 2019), which can be used to calculate seed length-to-width ratio (SLW) and seed weight per projection area (SWA). This procedure is simple, accurate, and has high throughput compared to manual measurements using Vernier calipers.

Natural selection of larger seeds in soybean has resulted in an accumulation of minor QTLs (Liu et al., 2007), and QTL mapping has provided insight into these evolutionary changes (Salas et al., 2006; Xu et al., 2011; Hina et al., 2020). Early, composite interval mapping (CIM) using Windows QTL Cartographer (Wang et al., 2006) or inclusive composite interval mapping (ICIM) using QTL IciMapping (Li et al., 2007) were applied to QTL mapping. However, the CIM and ICIM methods can only detect additive QTLs and additive–epistatic QTLs, while providing no QTL × environment information. In contrast, the mixed-model-based composite interval mapping (MCIM) of QTL Network can detect additive, epistatic, and QTL × environment interactions, thus providing more detailed QTL information (Yang et al., 2008).

Linkage mapping has been widely used in detecting quantitative trait loci (QTL) of soybean 100-SW (Stombaugh et al., 2004; Kato et al., 2014; Yang et al., 2019). To date, 304 QTLs have been identified for 100-SW in soybean; most are minor QTLs and the candidate genes have yet to be validated (Karikari et al., 2019). There are 29 QTLs for SL mapped to 13 chromosomes, 25 for SW on 13 chromosomes, and 18 for SLW on 11 chromosomes in SoyBase^1^.

Besides, genome-wide association studies (GWAS) has been also widely used to analyze soybean 100-SW and seed traits for natural populations (Zhang et al., 2016b; Li et al., 2019; Zhao et al., 2019; Qi et al., 2020). For example, a new seed size locus *SW9-1* was found through the GWAS study for 100-SW, SL and SW (Li et al., 2019). GWAS is powerful in detecting additive QTLs both for natural population and bi-parental population, but if epistatic QTLs are involved, new GWAS procedure is not available yet. Therefore, in the present study, the linkage mapping procedure MCIM of QTL Network will be considered for a recombinant inbred line (RIL) population.

Given the lower marker density in early genetic linkage maps, chromosome regions potentially harboring 100-SW and seed shape QTLs were too broad and often overlooked, leading to imprecise mapping that was not useful for identifying candidate genes for marker-assisted breeding. Recent advances in sequencing technology have led to the discovery of new markers and the generation of high-density molecular genetic linkage maps to detect 100-SW QTLs for annotated candidate genes in soybean (Karikari et al., 2019). Thus, a high-density molecular genetic map is a basic requirement for QTL fine mapping and candidate gene discovery (Zhang et al., 2016a; Wei et al., 2019).

100-seed weight and seed shape are heritable traits conferred by both major and minor genes (Zhao et al., 2019). For example, the *GmGA20OX* gene affecting SL, SW, and 100-SW encodes an enzyme involved in gibberellin synthesis (Lu et al., 2017), while the *Arabidopsis* homolog of *GmCYP78A10*, regulates SL, SW, seed thickness, and seed weight in soybean (Wang et al., 2015). The *SoyWRKY15a* gene associated with soybean seed volume and weight was identified through a combination of RNA sequencing and QTL mapping (Gu et al., 2017). However, the up-/downstream relationships of these genes and the mechanisms by which they regulate seed traits during plant development are unclear. The aim of the present study was to identify a QTL system for seed weight and morphology including additive, epistatic, and QTL × environment interactions of 100-SW, SL, SW, SP, and SA as well as SLW and SWA using an enhanced high-density genetic linkage map for a population of the RIL NJRISX. From the QTL system, candidate genes were annotated and validated using published RNA expression datasets. As seed weight and shape traits are interrelated, we speculated that their genetic constitutions may be somewhat overlapping, therefore, the genetic relationships among these traits were also examined.

## Materials and Methods

### Plant Materials and Field Experiments

A population of the RIL NJRISX was established from 155 F_2_-derived homozygous lines obtained by crossing “Su 88-M21” and “XYXHD”. The large-sized round seed of “Su 88-M21”, small-sized oval seed of “XYXHD” and the RIL population (NJRISX) derived from the cross were provided by the National Soybean Improvement Center of Nanjing Agricultural University. RILs along with the parents were tested in three environments: Jiangpu Experimental Station of Nanjing Agricultural University, Nanjing, Jiangsu Province (latitude 33°03′ N; longitude 118°63′ E) in June 2017 (17JP); Wanjiang Station of Nanjing Agricultural University, Dangtu County, Anhui Province (latitude 32°87′ N; longitude 117°56′ E) in June 2017 (17DT); and the same location in June 2019 (19DT). Each line was planted in a single row plot (length × width, 1 × 0.5 m) in a randomized complete block design with three replicates. Uniform agronomic practices were used in the experiments.

### Measurement of 100-Seed Weight and Seed Shape Traits

After harvesting, seeds were dried to a uniform moisture. Diseased, insect-infested, and physically damaged seeds were removed. Seed weight was measured using an electronic balance with 0.001-g accuracy. SL, SW, SP, and SA were measured from images using a high-speed camera (Model eloam-S1500A2; Shenzhen E-Loam Technology Co., Shenzhen, China). About 120 seeds per plot were collected and distributed evenly in the middle of the light-emitting diode backlight board of the equipment to obtain clear and complete images of the seeds. The board was calibrated and adjusted to an appropriate brightness according to the indoor light level. The equipment parameters were as follows: brightness, 64 cd/m^2^; contrast, 15; hue, 0; saturation, 43; clarity, 100; gamma, 100; white, balance 4600 auto, backlight contrast 0, power line frequency (anti-flicker) 50 Hz, focus 65, exposure −6; and scan size, 640 × 480 mm. The acquired images were processed using previously developed software (Ding et al., 2019). The image background was removed based on the “Otsu” threshold method to obtain the binary image of soybean seeds, and the adhesive soybean seeds in the binary map were segmented and counted based on the watershed transformation method. The sum of white pixels in each connected domain and the correction formula based on the freeman chain code algorithm is used to calculate seed area and perimeter, respectively. The second-order statistical moment is used to obtain the main axis direction of the soybean seeds and then the SL and SW was calculated by the extreme difference of the boundary point. Besides, this software can process hundreds of photos at a time and export the data to EXCEL. Data on the exact number of seeds on the backlight board, SL, SW, SP, and SA were directly obtained using the computer software and 100-SW was converted from seed number and seed weight values; SLW = SL/SW and SWA = 100-SW/SA were calculated. All seven seed weight and shape traits were measured in three replicates under three environmental conditions.

Before measuring the experimental seeds, in order to confirm that images obtained of a seed lot from a given plot were reproducible, two seed samples of the 138 lines were obtained from a single replicate and SL, SW, SP, and SA were measured under the same conditions using the same instrument. The correlation coefficients between the two measured values of SL, SW, SP, and SA were 0.95, 0.92, 0.93, and 0.94, respectively, indicating good consistency between the measurements from the same seed lot (Supplementary Table S1) and validating the utility of the imaging procedure used in this study.

### Statistical Analysis of Phenotypic Data

Data were analyzed using Excel 2016 software (Microsoft, Redmond, WA, United States). Analysis of variance (ANOVA) and correlation analysis were performed using SAS v9.4 software (SAS Institute, Cary, NC, United States). Heritability (*h*^2^) was calculated as

$$h^{2}=\frac{\sigma_{\text{g}}^{2}}{\sigma_{\text{g}}^{2}+\frac{\sigma_{\text{gy}}^{2}}{n}+\frac{\sigma_{e}^{2}}{n\,r}},$$

where σ^2^_*g*_, σ^2^_*gy*_, and σ^2^_*e*_ are genotype, genotype × environment interaction, and error variance estimated from the expected mean squares in ANOVA, respectively; *n* is the number of environments; and *r* is the number of replicates. The genotypic coefficient of variation (*GCV*) was calculated as *GCV* = σ_*g*_/μ, where μ is the mean value of the RIL population.

### Specific-Locus Amplified Fragment Sequencing (SLAF-seq) and Genetic Linkage Map Construction

In 2015, two parents and 155 progeny soybean lines were planted at Jiangpu Experiment Station. Genomic DNA was extracted from young leaves and SLAF-seq (Sun et al., 2013; Zhang et al., 2016a) was performed by the Biomarker Technologies Corporation (Beijing, China). Briefly, the soybean genome sequence (*G. max*, Wm82.a1. v1) was used as a reference to predict digestion sites. *Rsa*I and *Hae*III were used to digest the genomic DNA and the obtained fragments (SLAF tags of 364–414 bp) were processed for target selection. After passing library quality inspection, sequencing was performed with the HiSeq 2500 system (Illumina, San Diego, CA, United States). Rice (*Oryza sativa*)^2^ was processed in the same manner and served as the control for library construction and sequencing to determine whether the enzymes used in this experiment were activated or there were other quality problems in library construction. A total of 281.93 M reads were generated for the two parents and 155 recombinant inbred lines. After discarding low-quality reads, 247,821 SLAF tags were screened; of these, 207,180 and 212,958 were identified from female parent (Su 88-M21) and male parent (XYXHD), respectively, with sequencing depths of 41.56 and 44.04 fold, respectively. There were 150,515 SLAFs in the RILs with 7.75-fold coverage on average, corresponding to 1,473,406 reads. The reads of each identified sample were analyzed by clustering for SLAF tag screening. After removing low-quality reads, 52,988 tags that matched the parental separation pattern (Sun et al., 2013) were identified as polymorphic in the whole RIL population; these were screened according to SLAF tag filtering rules (Sun et al., 2013), yielding 9625 markers for linkage analysis.

Markers with high collinearity were removed for map construction, leaving 5351 SLAF markers. To obtain high-quality molecular tags, modified logarithm of odds scores between the tags were calculated and used for linkage grouping. HighMap software (Liu et al., 2014) was used to construct a genetic map for each linkage group. The software uses an efficient maximum likelihood estimation method to correct label classification based on the layout results, and after multiple cycles of layout correction–layout, a high-quality map is obtained. Map quality was evaluated in terms of co-linearity, genetic relationships, and monomer sources. The linkage map (Supplementary Figure S1) was drawn using the R-based software LinkageMapView (Ouellette et al., 2018).

### Mapping QTLs Conferring 100-Seed Weight and Seed Shape Traits

The MCIM function of QTL Network v2.0 was used to detect additive QTLs, additive × additive epistatic QTL pairs, and additive QTL × environment and epistatic QTL pair × environment interactions. The critical *F* value of MCIM was calculated with 1,000 permutation tests. The QTL effects were estimated by using the Monte Carlo Markov Chain method with 20,000 Gibbs sampler iterations and candidate interval selection; putative QTL detection and QTL effects were calculated with an experiment-wise type I error under *a* = 0.05 (Yang et al., 2007; Xing et al., 2012). The CIM method of Windows QTL Cartographer v2.5 software was used to perform CIM scanning of chromosomes to identify additive QTLs of soybean seed traits in different environments and verify the QTL mapping results of QTL Network v2.0 software. For CIM, the LOD significance threshold determined empirically using 1,000 permutation tests. Neighboring QTLs of different seed traits within the same support interval were grouped into a joint QTL segment (JQS). The 1-logarithm of the odds support (confidence) intervals were calculated using the QTL network procedure (Yang et al., 2007), which is defined by points on the genetic map at which the likelihood ratio has fallen from the maximum by a factor of 10 (Lander and Botstein, 1989).

Positive and negative additive effects indicated that the alleles were from “Su 88-M21” and “XYXHD” for all seed traits, respectively. Genetic contribution of the collective unmapped minor QTLs is equal to total genetic contribution minus variation explained by all detected additive and epistatic QTLs (Korir et al., 2011). Random error variation is equal to total phenotypic variation minus total genetic contribution, and variation explained by all detected additive QTL × environment and epistatic QTL× environment interactions (Xing et al., 2012).

### Annotation of Candidate Genes Conferring 100-Seed Weight and Seed Shape Traits

Candidate gene annotation was carried out for JQSs based on physical locations in SoyBase^3^. Gene ontology (GO) annotation v1.1 was downloaded from SoyBase^4^ and GO classification was based on clusterProfiler package (Yu et al., 2012) in R software (*p* value < 0.01, *q* value < 0.05) to identify terms related to seed weight and shape traits in soybean. To determine whether these genes are expressed in seeds, gene expression data of 14 soybean tissues at seven seed development stages (seed_10DAF, seed_14DAF, seed_21DAF, seed_25DAF, seed_28DAF, seed_35DAF, and seed_42DAF, where DAF stands for days after flowering) and 7 other tissues (young_leaf, flower, one.cm.pod, pod.shell.10DAF, pod.shell.14DAF, root, and nodule) (Severin et al., 2010) were downloaded from SoyBase^3^. Expression data from cultivated soybean were used as an approximation for analyzing the parents in the present study. The candidate genes were classified according to Protein Class using Protein ANalysis THrough Evolutionary Relationships (PANTHER)^5^ and annotated based on the National Center for Biotechnology Information (NCBI)^6^ and UniProt Protein^7^ databases to determine gene function.

## Results

### Phenotypic Variations (PVs) in 100-Seed Weight and Seed Shape Traits

**FIGURE 1:**
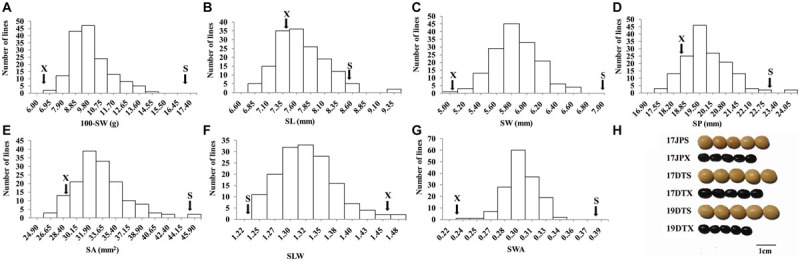
Frequency distribution of seed traits of the RIL population NJRISX. **(A–G)** Average frequency distribution (number of lines) for 100-seed weight (100-SW), seed length (SL), seed width (SW), seed perimeter (SP), seed projection area (SA), ratio of seed length-to-width (SLW) and seed weight per projection area (SWA). “S” and “X” represent the parents “Su 88-M21” and “XYXHD”, respectively. **(H)** Seed figure of the two parents “S” and “X” in the frequency distributions. Scale bar: 1 cm; 17JP, 17DT, and 19DT represent 2017 Jiangpu, 2017 Dangtu, and 2019 Dangtu, respectively, “S” and “X” represent the parents “Su 88-M21” and “XYXHD”, respectively.

The seed of the maternal parent “Su 88-M21” is round and large, while that of the paternal parent “XYXHD” is flat and small (Figure 1H); as these seeds differ significantly in terms of 100-SW, SL, SW, SP, SA, SLW, and SWA, it was possible to establish a RIL population with potential for genetic variation in these seven seed weight and shape traits (Table 1). Accordingly, the frequency distributions all showed a large variation (Figure 1 and Table 1), while transgressive segregation was observed for SL, SP, and SA in both directions (Figures 1B,D,E). In the joint ANOVA for multiple environments, there existed significant differences among Lines and Line × Env. interactions for all the traits except no significant differences among lines in SWA (Table 2). The phenotypic data from multiple environments were used to estimate heritability. The heritability of the seven traits ranged from 60.3 to 88.2% (Table 1). These results showed that further QTL constitution analysis for the traits except SWA would be meaningful.

**TABLE 1:** Descriptive statistics, broad-sense heritability (*h*^2^), and genotypic coefficients of variation (*GCV*) of seed traits in the recombinant inbred line population NJRISX and the parents “Su 88-M21” and “XYXHD”.

**Trait**	**Parent**		**NJRISX**					
	**P1**	**P2**	**Mean**	**Range**	***F*-Value**	***CV* (%)**	***h*^2^ (%)**	***GCV* (%)**
100-SW (g)	17.10	6.44	9.46	6.03–14.15	9.3	15.0	77.0	13.9
SL (mm)	8.58	7.47	7.55	6.62–9.27	13.9	3.5	84.2	5.4
SW (mm)	6.97	5.06	5.75	4.95–6.55	11.6	3.2	84.1	4.7
SP (mm)	23.00	18.58	19.52	16.97–23.73	13.6	3.6	88.2	3.5
SA (mm^2^)	44.63	28.50	32.42	24.90–45.69	13.6	6.5	84.5	10.1
SLW	1.23	1.47	1.31	1.22–1.47	12.5	2.0	84.1	5.4
SWA	0.38	0.23	0.29	0.22–0.34	8.2	14.6	60.3	4.6

*P1, maternal parent “Su 88-M21”; P2, paternal parent “XYXHD”; 100-SW, 100-seed weight; SL, seed length; SW, seed width; SP, seed perimeter; SA, seed projection area; SLW, ratio of seed length-to-width; SWA, seed weight per projection area.*

**TABLE 2:** Joint ANOVA of seed traits of the three environments of the recombinant inbred line population NJRISX.

**Source**	**100-SW**		**SL**		**SW**		**SP**		**SA**		**SLW**		**SWA**	
	***MS***	***F***	***MS***	***F***	***MS***	***F***	***MS***	***F***	***MS***	***F***	***MS***	***F***	***MS***	***F***
Env.	3719.38	102.9**	48.01	26.7**	12.20	11.1**	349.94	22.8**	2877.00	23.1**	0.1748	62.8**	3.5058	317.2**
Block (Env.)	31.86	11.4**	1.60	23.0**	1.03	30.8**	14.09	28.7**	112.57	25.0**	0.0011	1.6	0.0082	3.2**
Line	14.21	1.9**	1.71	6.3**	0.73	6.2**	11.47	6.2**	108.35	6.4**	0.0197	8.2**	0.0050	0.9
Line × Env.	7.40	2.7**	0.27	4.0**	0.12	3.6**	1.86	3.8**	17.10	3.8**	0.0024	3.4**	0.0055	2.2**
Error	2.81		0.07		0.03		0.49		4.49		0.0007		0.0025	

In the joint ANOVA, Env., Block (Env.), Line, Line × Env. were designated as random items. **Represents significance at 0.01 probability level. 100-SW, 100-seed weight; SL, seed length; SW, seed width; SP, seed perimeter; SA, seed projection area; SLW, ratio of seed length-to-width; SWA, seed weight per projection area. *MS*, Mean square; *F*, *F*-Value.

Of the seven seed traits, 100-SW, SL, SW, SP, and SA are of the first order and can be directly measured, while SLW and SWA are second-order traits that are calculated from first-order traits. To clarify their relationships, a correlation analysis was performed for these traits. The correlation coefficients of the five first-order traits ranged from 0.78 to 1.00, showing that they are closely related (Table 3). Notably, the correlation coefficient between SP and SA was approximately 1.00; additionally, the high correlation between 100-SW and the other four first-order traits (0.88–0.94) implied that they have a common genetic basis. For the two second-order traits, SLW – which is related to seed shape – was not correlated with 100-SW or SW, while SWA – related to seed volume weight – was not correlated with SLW, their other correlations with the other first rank traits were not high, therefore, are not attractive traits. In this case, the second-order trait SWA was neglected and excluded in the QTL mapping analysis.

**TABLE 3:** Pearson’s correlation analysis of seed traits of the recombinant inbred line NJRISX^†^.

**Traits**	**100-SW**	**SL**	**SW**	**SP**	**SA**	**SLW**
100-SW		2	4	5	5	0
SL	0.88**		1	2	2	0
SW	0.89**	0.78**		4	5	0
SP	0.93**	0.98**	0.89**		5	0
SA	0.94**	0.96**	0.92**	1.00**		0
SLW	0.16	0.49**	−0.16	0.31**	0.24**	
SWA	0.77**	0.44**	0.54**	0.49**	0.51**	−0.05

^†^The decimal values in the lower left corner represent the correlation between traits, and the integer values in the upper right corner represent the number of joint QTL segments shared between traits. Pearson correlation coefficients were calculated from the average of three environments. **Significant at a 0.01 probability level. 100-SW, 100-seed weight; SL, seed length; SW, seed width; SP, seed perimeter; SA, seed projection area; SLW, ratio of seed length-to-width; SWA, seed weight per projection area.

### Genetic Linkage Map and Genetics of 100-Seed Weight and Seed Shape Traits

A molecular genetic linkage map with 5351 SLAF-seq markers was constructed that spanned 3046.52 cM with an average intermarker interval of 0.57 cM. Chr9 harbored the most markers at 500 and Chr11 had the fewest at 80; the latter spanned the shortest distance at 106.38 cM, whereas markers on Chr9 covered the largest distance at 199.24 cM (Supplementary Figure S1 and Supplementary Table S2). By comparing the physical positions of markers on each chromosome, a consistent relationship between physical and genetic distances was observed on all chromosomes except Chr11 and Chr17, indicating that the genetic map was of good quality (Supplementary Figure S2).

A total of 42 additive and 18 epistatic QTL pairs were detected for the six seed traits, accounting for 24.9–37.5% and 2.5–7.2% of the PV, respectively; thus, a large part of the genetic variance (heritability minus total QTL contribution, 35.0–56.7%) was not explained by these QTLs, and were instead attributed to a collection of undetected minor QTLs (Table 4). In addition to these QTLs, the PV was explained by additive QTL × environment interaction (1.4–9.5%), epistatic QTL × environment interaction (0.4–0.8%), and random error (2.4–14.0%). Thus, the largest portion of the genetic variation was explained by additive QTLs, with epistatic QTL pairs accounting for only a small part of the genetic variation in the six seed traits. Identification of the unmapped minor QTLs, which collectively accounted for a relatively large portion of the genetic variation, depends on improvements in the precision and sensitivity of mapping procedures. In addition, there were both additive and epistatic QTL × environment interactions, but these explained only a small part of the PV. Overall, the genetic and gene × environment components of the six traits were similar.

**TABLE 4:** Contributions of quantitative trait loci (QTLs) and their interactions to phenotypic variation for seed traits in NJRISX (%).

**Traits**	**Genetic contribution**				**ADD.QTL × environment^e^**	**Epistatic QTL × environment^f^**	**Random error^g^**	**Total^h^**
	**Additive QTL^a^**	**Epistatic QTL^b^**	**Minor QTL^c^**	**Total^d^**				
100-SW	37.5 (48.7) (8)	4.5 (5.8) (3)	35.0 (45.5)	77.0	9.5	0.8	12.7	100
SL	31.9 (37.9) (7)	5.0 (5.9) (2)	47.3 (56.2)	84.2	5.6	0.5	9.7	100
SW	24.9 (29.6) (5)	2.5 (3.0) (2)	56.7 (67.4)	84.1	4.4	0.4	11.1	100
SP	32.9 (37.3) (8)	7.2 (8.2) (4)	48.1 (54.5)	88.2	8.7	0.7	2.4	100
SA	35.9 (42.5) (8)	6.7 (7.9) (4)	41.9 (49.6)	84.5	6.1	0.6	8.8	100
SLW	36.9 (43.9) (6)	3.8 (4.5) (3)	43.4 (51.6)	84.1	1.4	0.5	14.0	100

*^*a,b*^Additive and epistatic QTLs: variation explained by all additive and epistatic QTLs for this trait. Numbers in the first pair of parentheses in the “Additive QTL” and “Epistatic QTL” columns are the contributions of the QTL to total genetic variation, while those in second pair of parentheses are the numbers of QTL and QTL pairs, respectively. ^*c*^Minor QTL: (genetic contribution of the collective unmapped minor QTLs) = (total genetic contribution) – (variation explained by all detected additive and epistatic QTLs) (Korir et al., 2011). The numbers in parentheses are the contributions of minor QTL to the total genetic variation. ^*d*^Total genetic contribution. ^*e*^Additive QTL × environment: variation explained by all detected additive QTL × environment interactions. ^*f*^Epistatic QTL × environment: variation explained by all detected epistatic QTL × environment interactions. ^*g*^Random error = (total phenotypic variation)−(total genetic contribution)−(variation explained by all detected additive QTL × environment and epistatic QTL × environment interactions) (Xing et al., 2012). ^*h*^Total phenotypic variation.*

### Additive and Epistatic QTLs Conferring 100-Seed Weight and Seed Shape Traits

**FIGURE 2:**
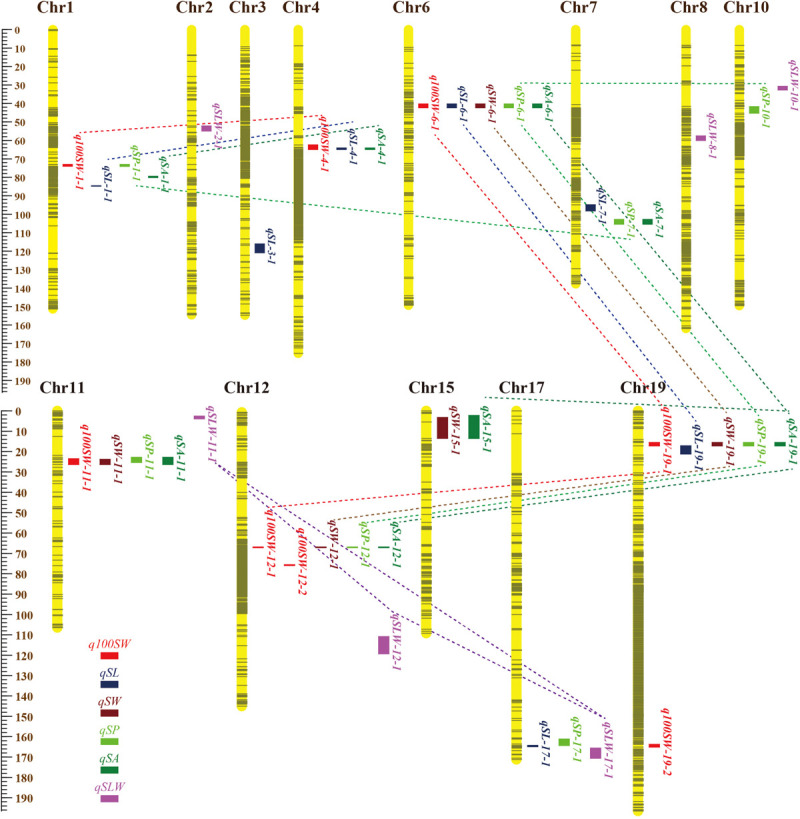
Locations and interactions of QTLs detected for seed traits in NJRISX. Different colorful dot lines represent the interaction between respective QTLs. The ruler on the left is shown in units of cM.

Table 5 shows information on each of the additive QTLs of the six seed traits. A total of 42 QTLs distributed on 13 chromosomes were identified by MCIM of the QTL network, of which 25 were also identified by CIM using Windows QTL Cartographer. For 100-SW, eight QTLs were identified on six chromosomes, each accounting for 1.8–8.2% of the PV (Figure 2 and Table 5). Two QTLs, *q100SW-6-1* and *q100SW-19-1* interacted with the environment and contributed 3.9 and 2.5% PV, respectively. Three QTLs have been previously reported in the literature (Supplementary Table S3). Seven QTLs on seven chromosomes were identified for SL, accounting for 3.3–6.8% of the PV; two have been previously reported. For SW, five QTLs on five chromosomes were identified, accounting for 2.3–8.4% of the PV; one QTL has been previously reported. For SP, eight QTLs on eight chromosomes were identified, accounting for 2.1–7.8% of the PV. Eight QTLs on eight chromosomes were identified for SA, accounting for 2.2–8.4% of the PV. For SLW, six QTLs on six chromosomes were identified, each accounting for 3.6–10.1% of the PV; two have been reported in the literature. Some of the identified additive QTLs interacted with each other to form significant epistatic QTL pairs involving all traits. There were three epistatic QTL pairs for 100-SW, two for SL, two for SW, four for SP, four for SA and three for SLW, with contributions to PV ranging from 0.6 to 3.0% for a single epistatic QTL pair (Figure 2 and Table 6) and 2.5–7.2% for a single trait. These low rates indicated that there was epistasis among genes governing seed weight and shape traits, although epistatic QTL pairs accounted for just a small part of the PV. Epistatic QTL pairs also interacted with the environment, with phenotypic contributions ranging from 0 to 0.7%; they also explained 0.4–0.8% of the PV for a single trait, indicating that they were relatively stable across environments.

**TABLE 5:** Quantitative trait locus (QTL) analysis for seed traits in NJRISX.

**QTL name^a^**	**Pos (cM)^b^**	**Support interval (cM)^c^**	**A^d^**	***p* value**	***h*^2^(a)%^e^**	***h*^2^(ae)%^f^**	**Cartographer^g^**	**Phy Pos (bp)^h^**
**100-seed weight (100-SW)**								
*q100SW-1-1*	**73.1**	73.0–74.0	–0.16	0.0055	1.8	0.9		35669867–35934002
*q100SW-4-1*	**64.8**	62.5–64.9	0.29	0.0000	5.8	0.5	1	16541907–16880516
*q100SW-6-1*	**41.3**	40.3–42.3	–0.40	0.0000	2.7	3.9	3	19981991–21026114
*q100SW-11-1*	**24.8**	23.5–26.4	0.26	0.0000	4.3	0.2	1,2	4899608–4989086
*q100SW-12-1*	**66.3**	66.2–66.5	1.41	0.0000	5.6	0.7	1,2	14741990–15675633
*q100SW-12-2*	75.1	74.9–75.2	–0.92	0.0000	4.8	0.8		19715891–20638016
*q100SW-19-1*	**16.6**	15.6–17.2	0.63	0.0000	8.2	2.5	3	44749755–45587224
*q100SW-19-2*	164.8	163.8–165.1	0.32	0.0000	4.3	0.0	2	4409800–6268959
**Seed length (SL)**								
*qSL-1-1*	84.7	84.6–84.8	–0.06	0.0014	3.9	0.6		42289253–42370498
*qSL-3-1*	116.8	116.1–120.8	–0.05	0.0045	3.3	0.1		38277812–39097580
*qSL-4-1*	**64.8**	64.1–64.9	0.08	0.0001	4.1	0.3		16541907–16880516
*qSL-6-1*	**41.7**	40.3–42.3	–0.15	0.0000	6.8	2.1	1,3	19374923–19981724
*qSL-7-1*	97.9	94.9–98.2	0.12	0.0000	5.3	0.4		7761878–8485600
*qSL-17-1*	**164.6**	164.3–164.9	–0.07	0.0004	3.7	0.8	1	1664060–1920098
*qSL-19-1*	18.2	17.2–21.2	0.12	0.0000	4.8	1.3	1	43190112–44749516
**Seed width (SW)**								
*qSW-6-1*	**41.3**	40.3–42.3	–0.12	0.0000	8.4	2.2	3	19981991–21026114
*qSW-11-1*	**25.4**	23.8–26.4	0.04	0.0007	3.2	0.1		4988816–5088557
*qSW-12-1*	**66.3**	66.2–66.5	0.11	0.0000	5.9	0.1	1	14741990–15675633
*qSW-15-1*	**8.5**	3.3–13.5	0.05	0.0007	2.3	0.0		1771617–2933099
*qSW-19-1*	**16.6**	15.6–17.2	0.11	0.0000	5.1	2.0	1,3	44749755–45587224
**Seed perimeter (SP)**								
*qSP-1-1*	**74.1**	73.0–74.1	–0.11	0.0111	2.4	0.8		35669867–35934002
*qSP-6-1*	**41.3**	40.3–42.3	–0.46	0.0000	7.8	3.2	1,3	19981991–21026114
*qSP-7-1*	**103.4**	102.8–105.4	0.29	0.0000	4.5	0.4	2	4586848–4858235
*qSP-10-1*	44.7	41.7–45.3	–0.18	0.0000	3.5	0.3		6104476–6376437
*qSP-11-1*	**23.8**	22.8–25.4	0.20	0.0000	4.5	0.4	1	4759306–4899857
*qSP-12-1*	**66.3**	66.2–66.5	0.29	0.0000	3.3	0.5	1	14741990–15675633
*qSP-17-1*	**163.6**	161.2–164.3	–0.16	0.0003	2.1	1.0	3	1981872–2379113
*qSP-19-1*	**16.6**	15.6–17.2	0.43	0.0000	4.8	2.1	3	44749755–45587224
**Seed projection area (SA)**								
*qSA-1-1*	79.7	79.5–80.1	–0.42	0.0027	2.7	0.9		39482451–40080311
*qSA-4-1*	**64.8**	64.1–64.9	0.68	0.0000	3.9	0.3		16541907–16880516
*qSA-6-1*	**41.3**	40.3–42.3	–1.42	0.0000	8.4	2.1	1,3	19981991–21026114
*qSA-7-1*	**103.4**	102.8–105.4	0.89	0.0000	5.2	0.2		4586848–4858235
*qSA-11-1*	**24.8**	22.8–26.4	0.53	0.0001	4.2	0.2	1	4899608–4989086
*qSA-12-1*	**66.3**	66.2–66.5	0.85	0.0000	3.9	0.5	1	14741990–15675633
*qSA-15-1*	**8.5**	2.3–13.6	0.57	0.0002	2.2	0.0		1771617–2933099
*qSA-19-1*	**16.6**	15.6–17.2	1.31	0.0000	5.4	1.9	3	44749755–45587224
**Ratio of seed length-to-width (SLW)**								
*qSLW-2-1*	52.9	52.2–54.9	–0.02	0.0000	10.1	0.2	1,2,3	16443169–38447603
*qSLW-8-1*	58.6	57.6–60.0	–0.01	0.0000	4.9	0.0		10129018–10431411
*qSLW-10-1*	31.6	30.7–32.7	–0.01	0.0000	4.6	0.2		4304666–4423053
*qSLW-11-1*	3.6	2.6–3.9	–0.01	0.0000	6.4	0.1		482225–716619
*qSLW-12-1*	113.2	110.2–118.6	–0.01	0.0011	3.6	0.6	3	34820304–35598025
*qSLW-17-1*	168.9	165.6–170.6	–0.01	0.0000	7.3	0.3	2	849675–672911

*^*a*^The QTL name is defined by the trait, chromosome number, and its order on the chromosome; the underline represents overlap with previous studies (detailed in Supplementary Table S3). ^*b*^Genetic position of the QTL; bold indicates location on joint QTL segment. ^*c*^Support (confidence) interval calculated using the QTL Network procedure (Yang et al., 2007). ^*d*^Estimated additive effect. ^*e*^Phenotypic variation explained by additive QTLs. ^*f*^Phenotypic variation explained by additive × environment interaction effects. ^*g*^The numbers 1, 2, and 3 indicate that the QTL was detected using QTL Cartographer v2.5 in 2017 Dangtu, 2017 Jangpu, and 2019 Dangtu, respectively. ^*h*^Physical position of QTL relative to that in soybean cultivar W82.a1.v.1.1.*

**TABLE 6:** Epistatic QTL pairs identified for seed traits in NJRISX.

**QTL-i**	**Position-i**	**QTL-j**	**Position-j**	**AA**	***p*-value**	***h*^2^(aa) (%)**	***h*^2^(aae) (%)**
**100-seed weight (100-SW)**							
***q100SW-1-1***	73.1	***q100SW-4-1***	64.8	–0.20	0.0006	1.3	0.0
***q100SW-6-1***	41.3	***q100SW-19-1***	16.6	–0.29	0.0000	2.2	0.7
***q100SW-12-1***	66.3	***q100SW-19-1***	16.6	0.21	0.0006	1.0	0.1
3						4.5	0.8
**Seed length (SL)**							
*qSL-1-1*	84.7	***qSL-4-1***	64.8	–0.09	0.0000	3.0	0.0
***qSL-6-1***	41.7	*qSL-19-1*	18.2	–0.08	0.0000	2.0	0.5
2						5.0	0.5
**Seed width (SW)**							
***qSW-6-1***	41.3	***qSW-19-1***	16.6	–0.05	0.0003	1.5	0.3
***qSW-12-1***	66.3	***qSW-19-1***	16.6	0.04	0.0025	1.0	0.1
2						2.5	0.4
**Seed perimeter (SP)**							
***qSP-1-1***	74.1	***qSP-7-1***	103.4	0.17	0.0001	2.2	0.1
*qSP-6-1*	41.3	*qSP-10-1*	44.7	0.13	0.0032	0.6	0.1
***qSP-6-1***	41.3	***qSP-19-1***	16.6	–0.21	0.0000	2.1	0.5
***qSP-12-1***	66.3	***qSP-19-1***	16.6	0.24	0.0000	2.3	0.0
4						7.2	0.7
**Seed projection area (SA)**							
*qSA-1-1*	79.7	***qSA-4-1***	64.8	–0.57	0.0000	1.7	0.0
***qSA-6-1***	41.3	***qSA-19-1***	16.6	–0.72	0.0000	2.3	0.4
***qSA-12-1***	66.3	***qSA-19-1***	16.6	0.53	0.0002	1.3	0.1
***qSA-15-1***	8.5	***qSA-19-1***	16.6	0.52	0.0009	1.4	0.1
4						6.7	0.6
**Ratio of seed length-to-width (SLW)**							
*qSLW-11-1*	3.6	*qSLW-12-1*	113.2	–0.01	0.0001	1.2	0.1
*qSLW-11-1*	3.6	*qSLW-17-1*	168.9	–0.01	0.0011	0.8	0.1
*qSLW-12-1*	113.2	*qSLW-17-1*	168.9	0.01	0.0000	1.8	0.3
3						3.8	0.5
**Total 18 epistasis QTL pairs, 2–4 pairs per each trait**						2.5–7.2	0.4–0.8

*QTL, quantitative trait locus/loci; *QTL-i* and *QTL-j*, epistatic QTL pairs (bold indicates location on joint QTL segment); *AA*, estimated additive × additive–epistatic effect; *h*^2^(aa), phenotypic variation explained by the additive × additive interaction; *h*^2^(aae), phenotypic variation explained by the additive × additive × environment interaction.*

The major QTL *qSLW-2-1* was located at 52.2–54.9 cM on Chr2, accounting for 10.1% of the PV, and was detected by Cartographer under all three environmental conditions. These seed trait additive QTLs interacted with the environment and their phenotypic contribution ranged from 0 to 3.9%; this is not a large proportion, it indicated that the identified QTLs were relatively stable.

### JQSs Related to 100-Seed Weight and Seed Shape Traits

A QTL of a trait may be located in the same chromosomal region as QTL(s) of other trait(s). These QTLs may be either different loci or the same locus (referred to as JQSs) due to random shifting. Of the 42 identified QTLs, 28 were located in nine JQSs on nine chromosomes (Table 7).

**TABLE 7:** Joint QTL segments and their interactions for seed traits in NJRISX*.

**Joint QTL segment^†^**	**QTL-i**	**Support interval**	**Joint QTL segment**	**QTL-j**
JQS-1	*q100SW-1-1*	73.0–74.0	JQS-4	*q100SW-4-1*
	*qSP-1-1*	73.0–74.1	JQS-7	*qSP-7-1*
JQS-4	*q100SW-4-1*	62.5–64.9	**JQS-1**	***q100SW-1-1***
	*qSL-4-1*	64.1–64.9		
	*qSA-4-1*	64.1–64.9		
JQS-6	*q100SW-6-1*	40.3–42.3	JQS-19	*q100SW-19-1*
	*qSL-6-1*	40.3–42.3		
	*qSW-6-1*	40.3–42.3	JQS-19	*qSW-19-1*
	*qSP-6-1*	40.3–42.3	JQS-19	*qSP-19-1*
	*qSA-6-1*	40.3–42.3	JQS-19	*qSA-19-1*
JQS-7	*qSP-7-1*	102.8–105.4	**JQS-1**	***qSP-1-1***
	*qSA-7-1*	102.8–105.4		
JQS-11	*q100SW-11-1*	23.5–26.4		
	*qSW-11-1*	23.8–26.4		
	*qSP-11-1*	22.8–25.4		
	*qSA-11-1*	22.8–26.4		
JQS-12	*q100SW-12-1*	66.2–66.5	JQS-19	*q100SW-19-1*
	*qSW-12-1*	66.2–66.5	JQS-19	*qSW-19-1*
	*qSP-12-1*	66.2–66.5	JQS-19	*qSP-19-1*
	*qSA-12-1*	66.2–66.5	JQS-19	*qSA-19-1*
JQS-15	*qSW-15-1*	3.3–13.5		
	*qSA-15-1*	2.3–13.6	JQS-19	*qSA-19-1*
JQS-17	*qSL-17-1*	164.3–164.9		
	*qSP-17-1*	161.2–164.3		
JQS-19	*q100SW-19-1*	15.6–17.2	**JQS-6**	***q100SW-6-1***
			**JQS-12**	***q100SW-12-1***
	*qSW-19-1*	15.6–17.2	**JQS-6**	***qSW-6-1***
			**JQS-12**	***qSW-12-1***
	*qSP-19-1*	15.6–17.2	**JQS-6**	***qSP-6-1***
			**JQS-12**	***qSP-12-1***
	*qSA-19-1*	15.6–17.2	**JQS-6**	***qSA-6-1***
			**JQS-12**	***qSA-12-1***
			**JQS-15**	***qSA-15-1***
Total 9	28 QTLs in JQSs		8 QTLs of (a)	11 pairs of (aa)

*Epistatic QTL pairs with a bold QTL is reciprocal and duplicated. ^†^Defined as neighboring QTLs within overlapped support (confidence) intervals, which were calculated using the QTL network procedure (Yang et al., 2007, originally in Lander and Botstein, 1989). QTL-i and QTL-j are an epistatic QTL pair composed of QTL-i and QTL-j. a, additive; aa, additive × additive interaction; JQS, joint QTL segment.

The nine JQSs were designated as JQS-1, -4, -6, -7, -11, -12, -15, -17, and -19 (Table 7) and were composed of 2, 3, 5, 2, 4, 4, 2, 2, and 4 QTLs, respectively, on Chr 1, 4, 6, 7, 11, 12, 15, 17, and 19, respectively. JQS-6 harbored five QTLs, the most among JQSs (Figure 2). The 28 QTLs conferring different seed traits constituted nine QTL segments.

Joint QTL segment-1 was located at 73.0–74.1 cM on Chr1, covering 1.1 cM and containing two QTLs: *q100SW-1-1* and *qSP-1-1*. JQS-4 was located at 62.5–64.9 cM on Chr4, covering 2.4 cM and containing three QTLs: *q100SW-4-1*, *qSL-4-1* and *qSA-4-1*. JQS-6 was located at 40.3–42.3 cM on Chr6, covering 2.0 cM and containing five QTLs. JQS-7 was located at 102.8–105.4 cM on Chr7, covering 2.6 cM and containing two QTLs. JQS-11 was located at 22.8–26.4 cM on Chr11, covering 3.6 cM and including *q100SW-11-1*, *qSW-11-1*, *qSP-11-1*, and *qSA-11-1*. JQS-12 was located at 66.2–66.5 cM on Chr12, covering 0.3 cM and containing four QTLs. JQS-15 was located at 2.3–13.6 cM on Chr15, covering 11.3 cM and containing two QTLs. JQS-17 was located at 161.2–164.9 cM on Chr17, covering 3.7 cM and containing two QTLs. JQS-19 was located at 15.6–17.2 cM on Chr19, covering 1.6 cM and containing four QTLs (Table 7). Among the nine JQSs, JQS-1, -4, -6, -11, -12, and -19 harbored 100-SW and seed shape QTLs whereas only the latter were present in JQS-7, -15, and -17 (Table 7 and Supplementary Table S4).

Among the nine JQSs, JQS-11 and -17 were independent of the others, whereas the remaining seven JQS interacted with other JQSs (epistasis) or else harbored epistatic QTL. Of these JQSs, JQS-19 interacted with JQS-6 and JQS-12 for multiple traits. Although there were no interactions among the other JQSs, interactions were observed at the individual QTL level. Thus, a single or multiple QTLs in a JQS may interact with a single or multiple QTLs in another JQS, while some QTLs are independent and do not interact with other QTLs. Among the 28 QTLs on nine JQSs, eight were independent; 11 epistatic QTL pairs were located on JQSs; three epistatic QTL pairs partly located on JQS, e.g., *qSL-6-1* located on JQS, while *qSL-19-1* isolated from JQS; and the remaining four epistatic QTL pairs were isolated from JQSs (Figure 2 and Tables 6, 7).

### Functional Annotation of Candidate Genes in the Nine JQSs

**FIGURE 3:**
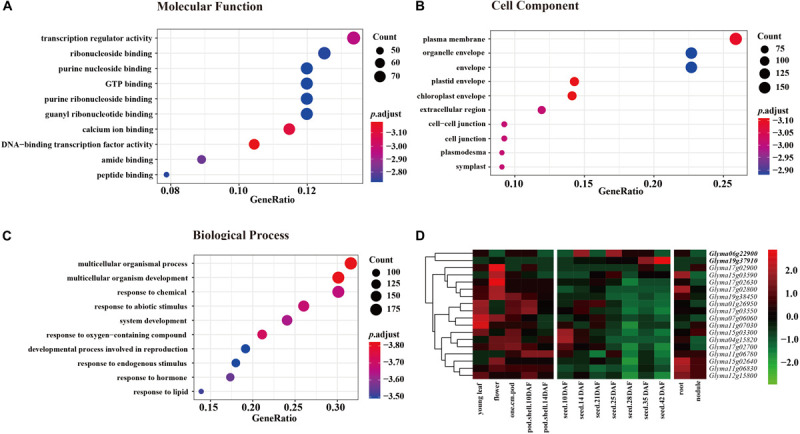
GO enrichment of 498 genes in three categories each with 10 groups and expression levels of 18 genes in 9 JQSs. For GO enrichment, *p* value < 0.01, *q* value < 0.05, and *p* value were corrected with Fisher’s exact test to obtain the *p*.adjust value, and were then transformed into –log10 (–log10 [*p*.adjust]). **(A)** GO enrichment of molecular function. **(B)** GO enrichment of cell component. **(C)** GO enrichment of biological process. **(D)** Heatmap of expression data for 18 candidate genes. Reads per kilobase of transcript per million mapped read values are normalized by row. Genes in boldface have high expression in seed tissues and low expression in other tissues.

Annotation of candidate genes in the nine JQSs was carried out using SoyBase^8^. JQS-1, -4, -6, -7, -11, -12, -15, -17, and -19 contained 6, 8, 57, 26, 48, 37, 149, 95, and 72 genes, respectively, for a total of 498 genes. In the GO enrichment analysis of 498 genes, in the molecular function category, most genes were enriched in terms related to activity and binding factors such as transcriptional regulator activity, ribonucleoside binding, and purine nucleoside binding (Figure 3A). In the cell component category, genes were mostly enriched in plasma membrane, organelle envelope, and envelope (Figure 3B). In the biological process category, the genes were enriched in multicellular organismal process, multicellular organism development, response to chemical, and other terms related to growth, development, and reproduction (Figure 3C).

Previously published gene expression data for soybean (Severin et al., 2010) were used for gene annotation. Of the 498 genes, we selected 294 located in the nine JQSs with expression in seed tissues; 99 were included in PANTHER protein classes, including 16 that were related to seed weight and morphology. Additionally, two genes were identified by analyzing protein functions in the gene database combined with literature searches (Supplementary Table S4). Ultimately, 18 genes that were mostly related to seed weight and shape were selected as candidate genes for further analysis, including 1, 1, 1, 1, 3, 1, 3, 5, and 2 candidate genes located in JQS-1, -4, -6, -7, -11, -12, -15, -17, and -19, respectively. Six of the genes were related to ubiquitin protein ligase; four to protein class of ribosomal protein; four to protein phosphatase; one (*Glyma19g37910*) to basic leucine zipper transcription factor; and one (*Glyma01g26950*) to tubulin; and one each encoded RING-H2 finger protein ATL52 and auxin response factor 18 (*Glyma04g15820* and *Glyma07g06060*, respectively) (Supplementary Table S4). The 18 genes have been shown to be related to seed size and shape traits in multiple plant species including soybean; in particular, *Glyma06g22900*, and *Glyma19g37910* have high expression in different seed tissues and low expression in other tissues (Severin et al., 2010), suggesting that they are important for seed development (Figure 3D and Supplementary Table S4). Thus, genes with similar functions in seed development are distributed in nine JQSs, which could account for the close relationship between seed weight and shape traits. In addition, some JQSs had a single candidate gene that conferred multiple traits whereas others had multiple candidate genes conferring several traits, indicating that some genes are pleiotropic and that multiple candidate genes may exist within a JQS.

## Discussion

### Genetic Basis of 100-Seed Weight and Seed Shape Traits

The results of this study provide an outline of the genetic structure of the RIL population NJRISX. We identified 42 additive QTLs that contributed 24.9–37.5% to the PV in seed weight and shape traits (100-SW, SL, SW, SP, SA and SLW), as well as 2–4 of 18 epistatic QTL pairs per trait for all six seed traits that contributed 2.5–7.2% of the PV. The remaining PV (35.0–56.7%) was explained by unmapped minor QTLs. The 28 additive and 11 epistatic QTL pairs were located in nine JQSs, suggesting that they were closely related and interacted. In addition, their interaction with the environment contributed 1.4–9.5% and 0.4–0.8% to the PV, respectively, although not large but significant.

Additive QTL is an important genetic component of seed weight and shape traits. Many QTLs associated with soybean seed weight and size have been identified (304 for 100-SW, 29 for SL, 25 for SW, and 18 for SLW in SoyBase^9^), but few have been examined in detail. Of the 42 QTLs detected in the present study, 8 were harbored in known loci (Supplementary Table S3) and the other 34 were identified for the first time; in 15 of these QTLs, each accounted for >5% of the PV.

Unmapped minor QTLs accounted for a considerable portion of the total PV, implying that more QTLs might be discovered by examining more lines in the population or more markers by improving the efficiency of the mapping procedure. Previous studies have demonstrated that restricted two-stage multi-locus genome-wide association study using single nucleotide polymorphism linkage disequilibrium block markers showed a superior performance to CIM, MCIM, joint inclusive CIM, and MLM-GWAS in mapping QTLs associated with days to flowering in soybean (Li et al., 2017; Pan et al., 2018). However, this procedure can identity additive QTLs and gene × environment QTLs but not epistatic QTL pairs. For a comprehensive analysis of the genetic structure of seed traits, both procedures can be used on the same dataset or a new mapping procedure can be applied.

### Correlation Among 100-Seed Traits and JQS Properties

The five first-order traits (100-SW, SL, SW, SP, and SA) were closely related with correlation coefficients ranging from 0.78 to 1.00, which was supported by the mapping results. Firstly, QTLs conferring different traits were located in the same segment, and could be the same QTL/gene with pleiotropic functions. Of the 42 QTLs, 28 were present in nine JQSs and 6, 3, 5, 7, and 7 QTLs were associated with 100-SW, SL, SW, SP, and SA, respectively. The number of JQSs shared between any two traits was highly consistent with their correlation coefficients (Table 3); that is, the number of shared JQSs forms the genetic basis of closely related seed traits. Secondly, along with additive QTLs, epistatic QTL pairs of traits were located on the same set of two JQSs, including JQS-6/-19, and JQS-12/-19; the parallel QTL interactions increased the correlation between the two traits. Additionally, additive effects of QTLs in the same JQS were in the same direction (positive or negative), suggesting a pleiotropic effect of the same QTL/gene or an aggregation of multiple QTLs/genes occurring in the same direction in the related traits. In other words, the allele effects contribution of the different QTLs in the same JQS are from a same parent. The number of QTLs and traits in the nine JQSs in the present study varied, indicating that each JQS has unique characteristics and functions. Genes with similar functions in seed development were separated in the JQSs while each JQS also harbored QTLs of genes with similar function, which could underlie the close association between seed traits.

The average segment length of overlapped confidence intervals in the nine JQSs was 3.2 cM (rang: 0.3–11.3 cM) – e.g., 11.3 cM for JQS-15 and 1.6 cM for JQS-19. The length depends on the number of QTLs linked at the same location (i.e., the density of markers in the segment). It is expected that by using higher-resolution genome-wide markers, more JQSs can be identified. From the present results, the JQSs can be grouped into three types based on whether individual QTLs in the JQS interact with others. (i) QTLs in the JQS all have epistatic interaction effects and interact with QTLs within the same JQS, resulting in a parallel relationship between traits. For example, every QTL in JQS-6 and -19 except those for SL (there was maybe a random shifting for *qSL-19-1*) had significant interactions with each other that appeared as interactions between the two JQSs, suggesting that the two segments had two interacting genes when they were actually the same QTL/gene with pleiotropic functions. JQS-12 and -19 also belong to this class. (ii) Some QTLs in JQSs (e.g., JQS-4, -7, and -15) show epistatic interactions but these do not occur in parallel between traits; or else different QTLs in same JQS interact with different JQSs, such as JQS-1. This class of JQSs contains QTLs with distinct properties (i.e., different QTLs for seed weight and seed shape traits) and hence, different genes. (iii) There is no epistatic interaction of QTLs in the JQS (e.g., JQS-11 and -17).

The above JQS results were observed in a set of mutually related traits, and would not be revealed if only a single trait was involved. When QTL mapping was performed for closely correlated traits, QTLs of different traits aggregated on neighboring or overlapping segments on the same chromosome (Liu et al., 2017), which are referred to as QTL hotspots (Zhang et al., 2019) or QTL clusters (Cai and Morishima, 2002). Because of limited marker density, QTL clusters were previously defined as regions with different QTLs located in the same or adjacent segments (Xu et al., 2011). While QTL clusters or hotspots have no defined thresholds, JQS is defined as a group of QTLs linked within confidence intervals; thus, using multiple related traits in JQSs can yield more precise and accurate results in the identification of QTLs/candidate genes. However, additional studies are needed to evaluate whether QTL grouping according to the support interval criterion of Lander and Botstein (1989) is appropriate.

## Conclusion

In this study, we examined the QTL system of six seed weight and shape traits (100-SW, SL, SW, SP, SA, and SLW) in the soybean RIL population NJRISX, and identified 42 additive QTLs and 18 epistatic QTL pairs accounting for 24.9–37.5% and 2.5–7.2% of the PV, respectively, with the remaining part of the PV (35.0–56.7%) attributable to unmapped minor QTLs. Notably, 28 additive QTLs and the 11 epistatic QTL pairs were concentrated in nine JQSs, indicating that seed weight and shape QTLs are closely related and interact; moreover, additive QTLs and epistasis QTL pairs interaction with the environment made a small but significant contribution to the PV (1.4–9.5% and 0.4–0.8%, respectively). Thus, the JQS is important for interpreting the close relationships among the six seed weight and shape traits, especially the five first-order traits. Our findings provide a whole picture of the genetic structure of soybean seed traits and demonstrate that examining a group of closely related traits can be more informative than analyzing individual traits.

## Data Availability Statement

We have uploaded our genetic linkage map information to GitHub (https://github.com/njau-sri/mengli-2020-ril).

## Author Contributions

ML, GX, JG, LC, JZ, and MR analyzed the data, prepared and analyzed the images, and wrote the manuscript. LC and YX performed phenotype analyses. JZ performed the bioinformatics analysis. ML, LC, XX, and YX planted soybeans in the field. ML, GX, WW, and JH performed genetic analyses. GX, WW, JH, and JG contributed reagents and materials and interpreted the results. All authors participated in manuscript drafting and revision, and approved the final version.

## Conflict of Interest

The authors declare that the research was conducted in the absence of any commercial or financial relationships that could be construed as a potential conflict of interest.

## Footnotes

1 https://www.soybase.org

2 http://rice.plantbiology.msu.edu/

3 https://www.soybase.org

4 https://www.soybase.org/genomeannotation/

5 https://www.soybase.org/

6 https://www.nlm.nih.gov/

7 https://www.uniprot.org/

8 https://www.soybase.org/genomeannotation/

9 https://www.soybase.org
