# Supplementary material for: Identification of Additive–Epistatic QTLs Conferring Seed Traits in Soybean Using Recombinant Inbred Lines
Source: Front Plant Sci. 2020 Dec 10;11:566056. doi: 10.3389/fpls.2020.566056 (PMC7758492; doi:10.3389/fpls.2020.566056)
Supplement: Supplementary file 1 [file Data_Sheet_1.doc]

Supplementary Material

**Supplementary Table 1.** Stability analysis of 2-repeat data for the 138 recombinant inbred line^†^

| Traits | Measure 1 | Measure 2 | Mean | RMSE | *CV* (%) | *r* value | *F* value |
| --- | --- | --- | --- | --- | --- | --- | --- |
| SL (mm) | 7.92 | 7.95 | 7.94 | 0.14 | 1.71 | 0.95** | 36.94** |
| SW (mm) | 5.96 | 6.01 | 5.99 | 0.13 | 2.10 | 0.92** | 20.47** |
| SP (mm) | 20.57 | 20.68 | 20.63 | 0.42 | 2.04 | 0.93** | 25.64** |
| SA (mm^2^) | 35.43 | 35.89 | 35.66 | 1.28. | 3.59 | 0.94** | 28.83** |

^†^Measure 1 indicates the first measurement for the 138 lines; Measure 2 indicates the second measurement of the same seed package from the same 138 lines of NJRISX under the same conditions. Mean, average of the 2 measurements for the 138 lines; RMSE, root mean square error; *CV*, error coefficient of variation; *r*, Pearson correlation coefficient; *F***,** *F* value among lines**.** ***P*<0.01.

SL, seed length; SW, seed width; SP, seed perimeter; SA, seed projection area.

**Supplementary Table 2.** Characteristics of the 20 constructed linkage groups

| Chr | Total  Marker | Total Distance (cM) | Average Distance (cM) | Max Gap (cM) |
| --- | --- | --- | --- | --- |
| 1 | 280 | 151.29 | 0.54 | 14.65 |
| 2 | 135 | 154.47 | 1.14 | 9.08 |
| 3 | 374 | 154.70 | 0.41 | 5.50 |
| 4 | 467 | 175.34 | 0.38 | 12.06 |
| 5 | 184 | 132.27 | 0.72 | 5.97 |
| 6 | 130 | 149.30 | 1.15 | 11.96 |
| 7 | 275 | 137.68 | 0.50 | 18.68 |
| 8 | 295 | 161.89 | 0.55 | 11.75 |
| 9 | 500 | 199.24 | 0.40 | 5.18 |
| 10 | 263 | 149.55 | 0.57 | 10.33 |
| 11 | 80 | 106.38 | 1.33 | 9.26 |
| 12 | 385 | 144.27 | 0.37 | 9.14 |
| 13 | 199 | 177.18 | 0.89 | 19.62 |
| 14 | 180 | 142.39 | 0.79 | 19.20 |
| 15 | 110 | 109.23 | 0.99 | 8.10 |
| 16 | 346 | 132.64 | 0.38 | 10.85 |
| 17 | 136 | 171.24 | 1.26 | 19.57 |
| 18 | 278 | 133.27 | 0.48 | 9.00 |
| 19 | 443 | 196.52 | 0.44 | 5.38 |
| 20 | 291 | 167.71 | 0.58 | 7.96 |
| Total/Average | 5351 | 3046.55 | 0.57 | 11.16 |

**Supplementary Table 3. Information on overlapped QTLs in references**

| QTL name | Physical Positon (bp)^a^ | References^b^ |
| --- | --- | --- |
| **100-seed weight (100-SW)** | | |
| *q100SW-6-1* | 19981991-21026114 | *Seed weight 16-2* (Funatsuki et al., 2005) |
| *q100SW-12-2* | 19715891-20638016 | *HSW-12-3* (Zhao et al., 2019) |
| *q100SW-19-1* | 44749755-45587224 | *Seed weight 17-1* (Stombaugh et al., 2004) *Seed weight 43-4* (Kuroda et al., 2013) |
| **Seed length (SL)** | | |
| *qSL-6-1* | 19374923-19981724 | *qSL-6-2_ZY_* (Hina et al., 2020) |
| *qSL-19-1* | 43190112-44749516 | *Seed length 1-10* (Salas et al., 2006) *Seed length 4-8* (Xu et al., 2011) |
| **Seed width (SW)** | | |
| *qSW-19-1* | 44749755-45587224 | *Seed width 1-7* (Salas et al., 2006) |
| **Ratio of seed length-to-width (SLW)** | | |
| *qSLW-2-1* | 16443169-38447603 | *Seed length to width ratio 1-2* (Xu et al., 2011) |
| *qSLW-10-1* | 4304666-4423053 | *Seed length to width ratio 1-6* (Xu et al., 2011) *Seed length to width ratio 1-7* (Xu et al., 2011) |

^a^ Physical position of QTL in relation to that in soybean cultivar W82.a1.v.1.1.

^b^ References with overlapped QTLs.

**Supplementary Table 4.** Information on 18 annotated candidate genes in 9 joint quantitative trait locus segments (JQS)

| Joint QTL segment | Gene ID | PANTHER protein class | Function annotation and reference |
| --- | --- | --- | --- |
| JQS-1 (100-SW, SP) | *Glyma01g26950* | Tubulin | Seed length in rice (Segami et al., 2012) |
| JQS-4 (100-SW, SL, SA) | *Glyma04g15820* | − | Width and weight of the seed in rice (Song et al., 2007) |
| JQS-6 (100-SW,SL,SW,SP,SA) | ***Glyma06g22900*** | **Ribosomal protein** | **Seed size and 1000-seed weight in tobacco (Tian et al., 2017)** |
| JQS-7 (SP, SA) | *Glyma07g06060* | − | Seed size and weight in *Arabidopsis*  (Schruff et al., 2005) |
| JQS-11 (100-SW, SW, SP, SA) | *Glyma11g06780* | Ubiquitin-protein ligase | Width and weight of the grain in rice (Song et al., 2017) |
|  | *Glyma11g06830* | Ubiquitin-protein ligase | Width and weight of the seed in rice (Song et al., 2007) |
|  | *Glyma11g07030* | Protein phosphatase | 100-seed weight and seed size in *Glycine max* (Lu et al., 2017) |
| JQS-12 (100-SW, SW, SP, SA) | *Glyma12g15800* | Ribosomal protein | Seed size and 1000-seed weight in *tobacco* (Tian et al., 2017) |
| JQS-15 (SW, SA) | *Glyma15g02640* | Protein phosphatase | Seed length, width and 100-seed weight in *Glycine max* (Lu et al., 2017) |
|  | *Glyma15g03300* | Ribosomal protein | Seed size and 1000-seed weight in *tobacco* (Tian et al., 2017) |
|  | *Glyma15g03590* | Ubiquitin-protein ligase | Width and weight of the grain in rice (Song et al., 2017) |
| JQS-17 (SL, SP) | *Glyma17g02630* | Ubiquitin-protein ligase | Width and weight of the grain in rice (Song et al., 2017) |
|  | *Glyma17g02700* | Protein phosphatase | Seed length, width and 100-seed weight in *Glycine max* (Lu et al., 2017) |
|  | *Glyma17g02800* | Ubiquitin-protein ligase | Width and weight of the seed in rice (Song et al., 2017) |
|  | *Glyma17g02900* | Protein phosphatase | Seed length, width and 100-seed weight in *Glycine max* (Lu et al., 2017) |
|  | *Glyma17g03550* | Ribosomal protein | Seed size and 1000-seed weight in *tobacco* (Tian et al., 2017) |
| JQS-19 (100-SW, SW, SP, SA) | ***Glyma19g37910*** | **Basic leucine zipper**  **Transcription factor** | **Seed length, width and 1000-seed weight in *Arabidopsis* (Cheng et al., 2014)** |
|  | *Glyma19g38450* | Ubiquitin-protein ligase | Width and weight of the seed in rice (Song et al., 2007) |

The traits of QTLs included in the JQS are shown in parentheses after the JQS; the genes in boldface indicate that they are highly expressed in seed tissues and have low expression in other tissues (Severin et al., 2010; **Fig. 3D**); and “−” indicates that the gene was not classified in the protein class.
