# Supplementary material for: Identification of Additive–Epistatic QTLs Conferring Seed Traits in Soybean Using Recombinant Inbred Lines
Source: Front Plant Sci. 2020 Dec 10;11:566056. doi: 10.3389/fpls.2020.566056 (PMC7758492; doi:10.3389/fpls.2020.566056)
Supplement: Supplementary file 2 [file Image_1.pdf]

Supplementary Material

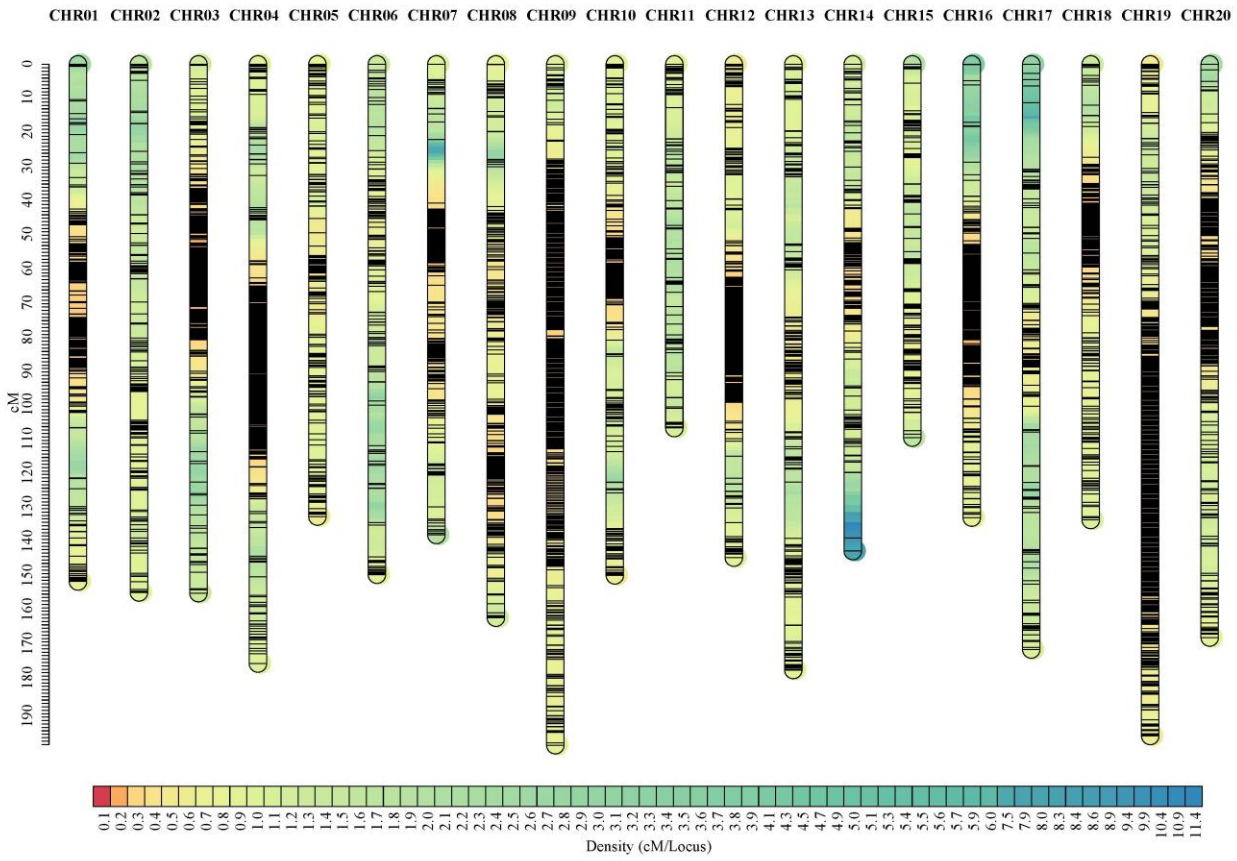

Supplementary Figure 1. High-density genetic linkage map of 20 chromosomes in the RIL NJRISX constructed based on SLAF-seq.

Marker positions are shown in centimorgans. Different colors represent different marker densities.

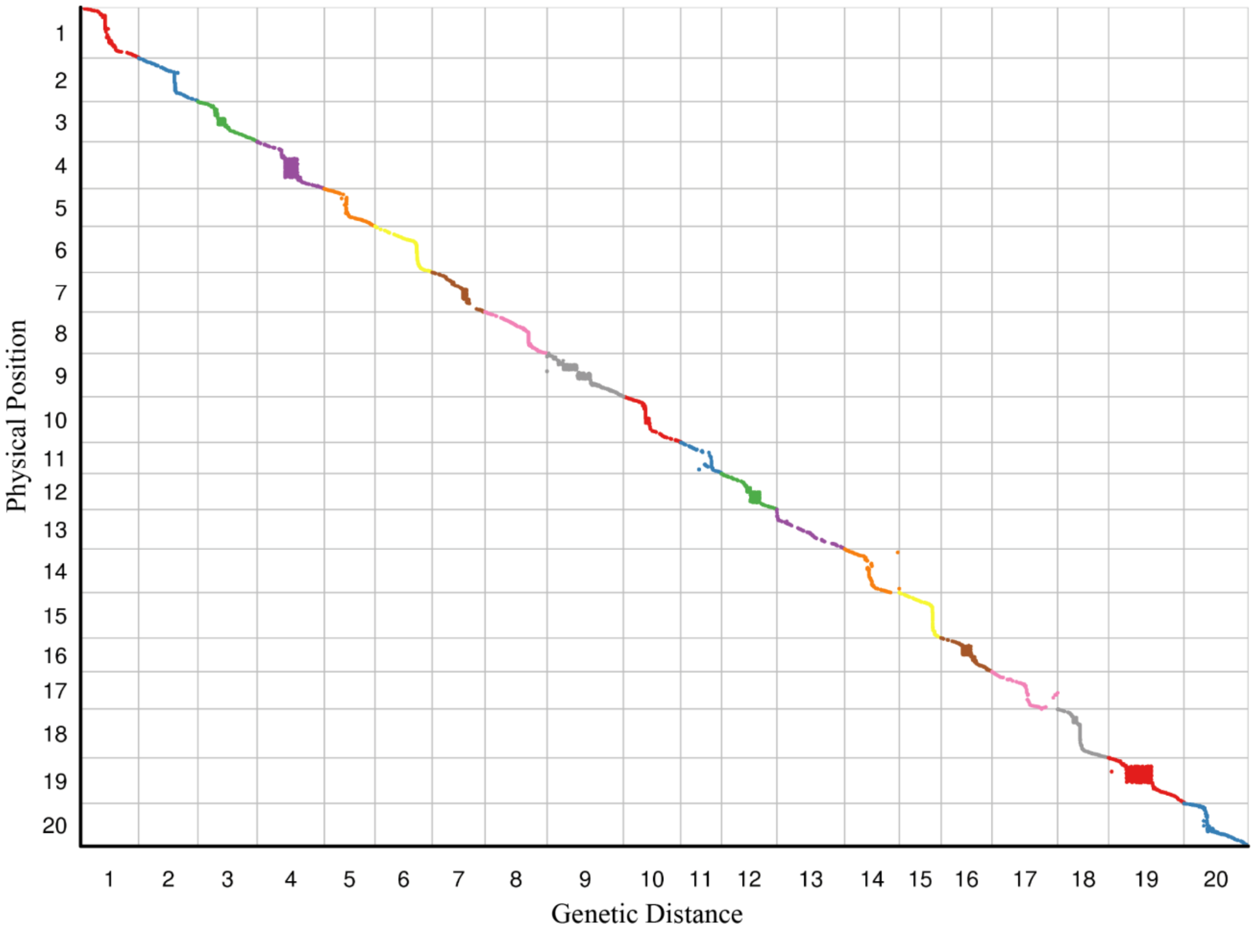

Supplementary Figure 2. Collinearity of 20 chromosomes with the soybean reference genome.
The x axis represents the genetic distance of soybean chromosomes, and the y axis represents linearity order of the physical position in the soybean genome. SLAF markers on the chromosomes are plotted as dots on the figure, with different colors indicating different chromosomes.
